# Knowledge, attitudes and preventive practices of primary health care professionals towards alcohol use: A national, cross-sectional study

**DOI:** 10.1371/journal.pone.0216199

**Published:** 2019-05-13

**Authors:** Esperanza Romero-Rodríguez, Luis Ángel Pérula de Torres, Fernando Leiva-Cepas, José Ángel Fernández García, Sara Fernández López, María Martín-Rabadán Muro, Francisco Camarelles Guillem, Ana Roldán Villalobos, for the Collaborative Group Alco-AP

**Affiliations:** 1 Maimonides Biomedical Research Institute of Cordoba (IMIBIC), Reina Sofia University Hospital, University of Cordoba, Cordoba, Spain; 2 Teaching Unit of Family and Community Medicine, Health District of Cordoba and Guadalquivir, Cordoba, Spain; 3 Preventive Activities and Health Promotion Program -PAPPS- (semFYC), Barcelona, Spain; 4 PAPPS Evaluation and Improvement Group (semFYC), Barcelona, Spain; 5 USBA Cerro Muriano. Brigada Guzmán ¨El bueno¨ X. Ministry of Defence, Cordoba, Spain; 6 Villarrubia Health Center, Andalusian Health Service, Cordoba, Spain; 7 Villanueva del Rey Health Center, Andalusian Health Service, Cordoba, Spain; 8 Can Misses Health Center, Ibiza, Spain; 9 Infanta Mercedes Health Center, Madrid, Spain; 10 Carlos Castilla del Pino Health Center, Andalusian Health Service, Cordoba, Spain; Northumbria University, UNITED KINGDOM

## Abstract

**Introduction:**

Primary care (PC) professionals' knowledge about alcohol use has been identified as one of the barriers PC providers face in their clinic. Both PC professionals’ level of training and attitude are crucial in the clinical practice regarding alcohol use.

**Objective:**

To evaluate the knowledge, attitude, and preventive practices of Spanish PC physicians and nurses towards alcohol use.

**Design:**

An observational, descriptive, cross-sectional, multi-center study.

**Methodology:**

Location: PC centers of the Spanish National Health System (NHS). Participants: PC physicians and nurses selected randomly from health care centers, and by sending an e-mail to semFYC and SEMERGEN members. Healthcare providers completed an online survey on knowledge, attitude, and follow-up recommendations for reducing alcohol intake. A descriptive, bivariate, and multivariate statistical analysis was conducted (p<0.05).

**Results:**

Participants: 1,760 healthcare providers completed the survey (75.6% [95% CI 73.5–77.6] family physicians; 11.4% [95% CI 9.9–12.9] medical residents; and 12.5% [95% CI 10.9–14.1] nurses), with a mean age of 44.7 (SD 11.24, range: 26–64, 95% CI: 47.2–48.2). Knowledge was higher in family physicians (p<0.001), older professionals (Spearman's r = 0.11, p<0.001), and resident trainers (p<0.001). The PC professional most likely to provide advice for reducing alcohol use was: a nurse (p <0.001), female (p = 0.010), between 46 and 55 years old (p <0.001).

**Conclusions:**

PC providers’ knowledge and preventive practices regarding alcohol use are scarce, hence specific training strategies to increase their knowledge and improve their attitude and skills with regard to this health problem should be considered a healthcare policy priority.

## Introduction

Alcohol use is a major public health problem [1]. Globally, it was the seventh leading risk factor for premature death and disability in 2016, and nearly three million deaths were attributed to its consumption [2]. Currently, harmful alcohol use contributes substantially to the global burden of disease, accounting for 5.1% of the morbidity and injury burden worldwide, measured in terms of disability-adjusted life years (DALYs) [3].

According to the latest alcohol and drug survey (EDADES, *Encuesta Domiciliaria sobre Alcohol y Drogas*) conducted in Spain [4], alcohol was the most widely consumed psychoactive substance (77.6%) in 2015. Although alcohol use indicators have remained quite stable since 2005, there has been an increase of binge drinking, which represented 17.9% of the population aged 15 to 64 years. Furthermore, the percentage of hazardous drinking in the Spanish population was 4.5%, and about 0.4% of the surveyed population could be labeled as having a possible dependence on alcohol.

At present, the criteria to define a pattern of at-risk alcohol use are clearly established in the Spanish Program for Prevention and Health Promotion Activities (PAPPS, *Programa de Actividades Preventivas y de Promoción de la Salud*), an initiative of semFYC, the Spanish Society of Family and Community Medicine, which is a national benchmark in Primary Care (PC) prevention [5]. The program defines the concept of at-risk alcohol use as an intake of over 2.0–2.5 standard drinks per day or over 17 standard drinks per week for women, and over 4 standard drinks per day or over 28 standard drinks per week for men, considering one standard drink as the equivalent to 10 grams of pure alcohol.

Health professionals’ knowledge and attitudes towards preventive strategies aimed at reducing alcohol use represent a public health priority [6]. A survey completed in European Union countries [7] found a direct relationship between the PC professionals’ level of training to handle alcohol-related problems and the number of patients treated. Similarly, Spanish studies [8] [9] conducted in Catalonia revealed that healthcare professionals’ knowledge on the approach to alcohol use was low, mainly due to the lack of training in preventive health.

Considering the social and health consequences arising from alcohol consumption and the low level of preventive actions accomplished in the PC setting, it seems justified to promote the implementation of alcohol prevention programs in this setting [10]. Previous studies [11] [12] highlighted the role played by PC professionals in the implementation of preventive strategies on alcohol use; hence, the knowledge, attitude, and experiences that they present about the preventive recommendations on alcohol consumption acquire special relevance.

Currently, the last PAPPS update recommends screening for at-risk alcohol consumption using the AUDIT (Alcohol Use Disorders Identification Test) questionnaire at least every 2 years for people over 14 years old [13]. Based on the score obtained, alcohol management strategies vary according to the level of risk identified: health education if the AUDIT score is below 8; simple advice if it is from 8 to 15; simple advice plus brief intervention if AUDIT is between 16 and 19; and referral for alcohol dependence assessment if the AUDIT score is 20 or more [14].

Despite global scientific evidence about alcohol management in the PC setting, only a few local studies have addressed the knowledge, attitude, and practices of PC professionals regarding alcohol prevention strategies in Spain [15] [16]. Therefore, a nationwide estimation of the knowledge, attitude, and practices of Spanish PC professionals on the management of at-risk alcohol use is required. We hypothesized that the level of knowledge of healthcare professionals regarding alcohol consumption in the PC setting is low. Likewise, few professionals offer preventive recommendations about alcohol use in their clinical practice.

Based on the above hypotheses, the objectives of this study are:

- To know the level of knowledge that PC healthcare professionals have concerning the PAPPS recommendations on alcohol use.
- To explore their clinical approach to patients with at-risk alcohol use, and to assess to what extent they offer preventive advice based on the PAPPS guidelines.
- To explore the relationship between sociodemographic and occupational variables of these professionals and the knowledge, implementation, and dissemination of preventive recommendations on alcohol use.

## Methodology

A nationwide observational, descriptive, cross-sectional, multi-center study was designed. The study population was formed by family physicians, nurses, and family medicine residents. The geographical scope included PC centers and Clinics of the Spanish National Health System (NHS). The study lasted 24 months, with a recruitment period running from August 2014 to August 2016 and was approved by the Clinical Ethics Committee at Reina Sofia University Hospital.

The selection criteria were: To be a PC professional (family physician, nurse or family medicine resident) of the NHS, and to give the consent to participate in the study.

The sample size of the study was calculated assuming an alpha error of 5%, accuracy of 3%, and a proportion of 50%. Consequently, it was necessary to include at least 1,068 healthcare professionals.

The study population was recruited in three ways:

1. Through participants from a previous study (CECC-AP study) [17], who were recruited through PAPPS and the semFYC Communication & Health Group. This included 240 professionals from 110 healthcare centers and clinics in Spain. Once the intention to participate was confirmed, the professionals completed the survey and were encouraged to invite other PC professionals from their centers to join this research. Thus, opportunistic and snowballing techniques were used.
2. By emailing members in the semFYC and SEMERGEN (Spanish Society of Primary Care Physicians) databases (16,474 and 8,000 members respectively) and uploading the study survey to their websites, making it freely available for anyone who wished to complete it.
3. Through stratified random sampling of NHS healthcare centers, according to the number of centers in each Spanish region. An email was sent to healthcare center managers, inviting both them and the other members of the PC team to participate, using a snowballing technique. The sample was obtained from the catalog developed by the Spanish Ministry of Health [18]. According to the 2014 database, there were 2,997 healthcare centers and 10,168 PC clinics, with an estimated number of 33,482 physicians working in public PC. Considering that 75% of the selected centers would’ve liked to collaborate in the study and an average of four healthcare professionals per health care center and two per local clinic would’ve liked to participate, a sample of at least 430 local healthcare centers and clinics was deemed necessary.

The information from each healthcare professional was obtained through a questionnaire prepared at the Family and Community Medicine Teaching Unit of Cordoba, in collaboration with the PAPPS Evaluation Group. The questionnaire was subjected to a process of consensus, apparent logic, and content validation (face validity). This questionnaire was developed for anonymous online self-completion (see: https://docs.google.com/forms/d/e/1FAIpQLSdrbHGqfwbHzB0NADEuDIVEdkzprA5wXVKr5oS_yD7wfIisZQ/viewform) with prior informed consent from each participant.

The variables analyzed in the survey included socio-demographic and occupational characteristics, knowledge of the PAPPS guidelines for alcohol use prevention, training received, adherence to PAPPS recommendations, and preventive practices and attitudes towards at-risk alcohol use.

The main study indicators were: knowledge of the PAPPS guidelines regarding the approach to alcohol, attitudes and practices focused on alcohol prevention, and the perception of the follow-up of their preventive advice for reducing alcohol intake.

STATISTICAL ANALYSIS: Data from surveys was automatically recorded into Google Forms by the participants and subsequently, exported to an Excel spreadsheet and statistically analyzed with SPSS v.17 and EPIDAT v.3.1 software.

Descriptive statistics of the variables were developed and 95% Confidence Intervals (95% CI) were calculated for the main study indicators. A bivariate analysis was then conducted to verify the relationship between independent variables and questionnaire responses (Spearman’s correlation coefficient, Chi-square test, comparison of mean tests like Student T-test and ANOVA test, after checking normality [Kolmogorov-Smirnov test], bilateral contrasts, p≤0.05). Finally, a multivariate analysis (multiple linear regression) was conducted to check socio-demographic occupational, and healthcare factors, which were independently associated with the study population's level of knowledge and personal and professional attitude to alcohol.

## Results

1,760 PC health care professionals completed the survey. Participants were predominantly female (62.9%; 95% CI: 60.6–65.2) and had a mean age of 44.7 years (SD 11.24; range: 26–64 years; 95% CI: 47.17–48.22). Two participation peaks were observed at 29 and 57 years; 75.6% (95% CI: 73.5–77.6) of the participants were family physicians, 11.4% (95% CI: 9.9–12.9) were medical residents, and 12.5% (95% CI: 10.9–12.4) were nurses; they had a mean work experience of 14.10 years (SD 10.55; range: 1–39, 95% CI: 13.60–14.59). Additionally, 33.4% (95% CI: 31.2–35.6) of the family physicians were trainers (S1 Table).

With regard to membership in scientific societies, 63.5% (95% CI: 61.2–65.7) were linked to semFYC; 26.8% (95% CI: 24.7–28.9) to SEMERGEN; 4.5% (95% CI: 3.5–5.5) to SEMG; and 1.2% (95% CI 0.7–1.7) to ASANEC. The response rate obtained in the study, considering membership in scientific societies, was 6.3%. Regarding assignment to specific prevention programs, such as the PAPPS, 25.9% (95% CI 23.8–28) of the professionals declared they were assigned to it.

In the analysis of overall knowledge on alcohol use, it was observed that the average of questions answered correctly by healthcare professionals was 2.68 ± 1.86 (95% CI 2.59–2.76; limits: 0 to 6), median: 2 (interquartile range: 3–6), up to 6 questions included (Table 1). Professionals’ age and work experience were directly correlated to their level of knowledge (Spearman's r = 0.11, p<0.001, and r = 0.14, p<0.001, respectively). Similarly, statistically significant relationships were obtained according to type of profession, age, and being a resident trainer, as shown in S2 Table. Family doctors, older physicians, and resident trainers showed the highest levels of knowledge. The multivariate analysis of the level of knowledge and the socio-demographic and occupational variables reveals a statistical relationship between PC professionals’ knowledge and being a resident trainer, having work experience, and having membership to PAPPS (Table 2).

**Table 1:** Knowledge of PC professionals regarding approach to alcohol.

Knowledge assessed	n (%)	95% CI
**Overall knowledge of the approach to alcohol use**^**a**^		
-Alcohol role as a risk factor in premature death and disease	1,033 (59.1)	56.4–61.0
-Concept of Standard Unit	915 (52.3)	49.7–54.3
-Concept of at-risk alcohol use		
Applied to men	873 (49.4)	47.3–51.9
Applied to women	612 (35.0)	32.5–37.0
-Concept of binge drinking		
Applied to men	630 (36.3)	33.6–38.0
Applied to women	624 (35.7)	33.2–37.7
**Knowledge of PAPPS recommendations on alcohol use**		
-Knowledge of PAPPS recommendations on alcohol use	27.5	25.4–29.6
-Knowledge of health advice on responsible alcohol use recommended by PAPPS	77.7	75.7–79.6
- Knowledge of alcohol quantification questionnaires recommended by PAPPS	62.7	60.4–65.0
- Knowledge of alcohol quantification questionnaires recommended by WHO/PAPPS		
Don't know	579 (32.9)	30.7–35.1
CAGE	566 (32.2)	30.0–34.3
AUDIT	386 (22.0)	20.0–23.9
MALT	99 (5.6)	4.5–6.7
Other	130 (7.4)	6.2–8.6
-Knowledge of patient follow-up recommended by PAPPS after detection of at-risk alcohol use	67.0	64.8–69.2
-Knowledge of criteria recommended by PAPPS for referral to a specialist	41.5	39.2–43.8

^a^Correctly answered survey questions; 95% CI: 95% confidence interval

**Table 2:** Variables associated with the level of knowledge on the approach to alcohol consumption through multivariate analysis, adjusted for age, sex, and profession.

Socio-demographic and occupational variables															
Knowledge	Age (years)				p-value ^a^	Sex		p-value ^a^	Profession			p-value ^a^	Resident trainer		p value ^a^
	Less than 35 n (%)	36–45 n (%)	46–55 n (%)	56 or more n (%)		Men n (%)	Women n (%)		Physicians n (%)	Residents n (%)	Nurses n (%)		Yes n (%)	No n (%)	
**Health advice for responsible alcohol use**	313 (65.9)	344 (79.6)	360 (84.5)	341 (82.2)	0.001	869 (79.1)	489 (75.3)	0.192	1,070 (80.9)	120 (57.7)	168 (77.1)	0.001	504 (86.3)	854 (73.4)	0.001
**Use of alcohol quantification questionnaire**	233 (49.1)	271 (62.7)	302 (70.9)	290 (69.9)	0.001	399 (61.5)	697 (63.4)	0.701	866 (65.5)	90 (43.3)	140 (54.5)	0.001	428 (73.3)	668 (57.4)	0.001
**Follow-up after detecting at-risk alcohol use**	270 (56.8)	309 (71.5)	332 (77.9)	311 (74.9)	0.001	449 (69.2)	773 (70.3)	0.862	962 (72.8)	112 (53.8)	148 (67.9)	0.001	467 (80)	755 (64.9)	0.001
**Referral to a specialist**	185 (38.9)	186 (43.1)	165 (38.7)	190 (45.8)	0.001	465 (42.3)	726 (41.3)	0.120	559 (42.3)	70 (33.7)	97 (44.5)	0.005	241 (41.3)	376 (41.7)	0.001

Coefficient of Determination R^2^ = 0.153; Overall statistic of the model F = 8.364 (p<0.001)

Focusing on the knowledge of the PAPPS guidelines about alcohol use, 27.5% (95% CI: 25.4–29.6) of PC professionals stated they knew the PAPPS recommendations and 77.7% (95% CI: 75.7–79.6) indicated they had knowledge of health advice for responsible alcohol use (Table 1). Table 3 shows the analysis of PC professionals’ knowledge concerning the advice transmitted by PAPPS; data are statistically significant in favor of family doctors, professionals aged 46–55, and resident trainers.

**Table 3:** Knowledge of PC professionals on PAPPS recommendations according to socio-demographic and occupational characteristics.

Variables	Beta coefficient	p-value
**Age**	-0.010	0.828
**Sex**	0.0014	0.580
**Profession**	0.0014	0.576
**Resident trainer**	0.052	0.046
**Work experience**	0.078	0.003
**Affiliated to PAPPS**	0.131	<0.001

^a^ The p-values were obtained using the Chi-square test.

Regarding the training that healthcare professionals received on the approach to alcohol use, 67.9% (95% CI: 67.5–71.8) reported that they had received no specific training in the last five years, 29.5% (95% CI: 27.4–31.7) claimed to have received basic training on this topic, and 2.6% (95% CI: 1.8–3.3) received average training. There was a statistical significance between the level of training of PC professionals and their age (p = 0.003), trainer status (p = 0.015), and work experience (p<0.001). A lower level of training was observed in those professionals older than 46 years who were not trainers and who had more work experience.

After a diagnosis of alcohol dependence syndrome, 61.9% (95% CI: 59.6–64.2) of PC professionals opted either for referral or treatment, depending on the patient's context; 24.0% (95% CI: 22.0–26.0) referred the patient to the drug dependence department; 1.6% (95% CI: 0.1–0.22) prescribed treatment by themselves; 5.5% (95% CI: 4.4–6.6) referred the patient to the psychiatry department; and 7.0% (95% CI: 5.8–8.2) claimed that they had not detected patients with this syndrome.

According to the clinical practice of PC professionals on the systematic examination of alcohol use, 29.0% (95% CI: 26.9–31.1) acknowledged doing this examination at their clinic, and 13.1% (95% CI: 11.5–14.7) stated they used quantification questionnaires when they suspected at-risk alcohol use (Table 4). In relation to the regular practice of the advice to abstain from alcohol use, data reveals that 73.2% (95% CI: 71.1–75.2) of PC professionals claimed to give this advice to pregnant women and 51.4% (95% CI: 49–53.7) gave it to patients who used dangerous machinery or drove motor vehicles.

**Table 4:** Practice of PC professionals regarding their approach to alcohol.

Practice of PC Professionals Regarding Their Approach to Alcohol	n (%)	95% CI
Systematic examination of alcohol use	29.0	26.9–31.1
Alcohol use recorded in computerized clinical history	85.6	83.9–87.2
Alcohol use quantified in computerized clinical history	75.0	73.0–77.0
Use of alcohol-use calculator in the computerized clinical history	56.2	53.9–58.5
Alcohol use quantification questionnaires	13.1	11.5–14.7
Advice on reducing alcohol use	45.5	43.1–47.8
Advice on alcohol abstinence for pregnant women	73.2	71.1–75.2
Advice on alcohol abstinence for machine operators and drivers	51.4	49.0–53.7

95% CI: 95% Confidence interval

The practice of PC professionals according to socio-demographic and occupational variables (Table 5) reveals statistically significant relationships according to age, sex, profession, being a resident trainer, and work experience. Nurses (p<0.001) who were female (p = 0.010) and aged between 46 and 55 (p<0.001) were the most likely to develop these practices.

**Table 5:** Practice of PC professionals in their approach to alcohol according to sociodemographic and occupational characteristics.

Sociodemographic and occupational variables															
Clinical practice	Age (years)				p-value ^a^	Sex		p-value ^a^	Profession			p-value ^a^	Resident trainer		p-value ^a^
	Less than 35 n (%)	36–45 n (%)	46–55 n (%)	56 or more n (%)		Men n (%)	Women n (%)		Physicians n (%)	Residents n (%)	Nurses n (%)		Yes n (%)	No n (%)	
**Systematic examination**	104 (21.9)	121 (28)	150 (35.2)	131 (31.6)	0.003	154 (23.7)	352 (31.0)	0.001	327 (28.1)	46 (22.1)	88 (40.3)	0.001	192 (32.9)	314 (27.0)	0.001
**Use of quantification questionnaire**	49 (10.3)	56 (12.9)	64 (15)	60 (14.4)	0.006	75 (14.6)	154 (14.0)	0.001	171 (13.0)	19 (9.1)	39 (17.9)	0.006	104 (16.1)	135 (9.1)	0.001
**Advice on reducing use**	184 (38.8)	196 (45.4)	208 (48.8)	208 (50.2)	0.001	265 (40.5)	521 (48.7)	0.010	613 (21.9)	75 (36.1)	108 (49.5)	0.001	279 (47.8)	517 (44.4)	0.058
**Advice on abstinence for pregnant women**	334 (70.3)	308 (71.3)	327 (76.7)	310 (74.7)	0.808	469 (72.3)	810 (73.7)	0.376	988 (74.7)	228 (71.1)	143 (65.6)	0.002	431 (73.8)	848 (72.8)	0.392
**Advice on abstinence for machine operators and drivers**	214 (45)	204 (47.2)	240 (56.3)	241 (58.0)	0.031	469 (72.3)	810 (73.7)	0.195	703 (53.2)	92 (44.3)	104 (47.7)	0.064	309 (52.9)	590 (50.6)	0.264

^a^ The p-values were obtained using the Chi-square test.

In relation to PAPPS guidelines for clinical practice, 27.5% (95% CI: 25.4–29.6) of PC professionals declared they usually adhere to these recommendations and 54.0% (95% CI: 51.7–56.3) claimed to apply them only on some occasions. Overall, the perception of professionals regarding adherence to preventive recommendations for reducing alcohol intake in those patients who had at-risk use was 60.0% (95% CI: 57.7–62.3).

## Discussion

This study is the first nationwide analysis focused on the knowledge, attitude, and preventive practice of Spanish healthcare professionals towards alcohol use in the PC setting. Our results indicate a low level of PC provider’s knowledge and specific training on the management of these patients. Consequently, the level of preventive practices on alcohol use among PC professionals is scarce. Therefore, our findings could be considered in order to promote the implementation of training programs on alcohol use targeted to PC providers.

Currently, PC professionals’ knowledge on alcohol screening and brief interventions has been identified as one of the major obstacles they face in their clinical practices. In accordance with our results, the studies of Nilsen [19] and Johnson [20] revealed a low level of knowledge by healthcare professionals regarding alcohol use in their clinical practice. Similarly, previous European studies, such as PHEPA [21] (Primary Healthcare European Project of Alcohol), developed by the European Commission and the Department of Health of Catalonia, pointed to the need to promote alcohol prevention activities in the PC setting. In this context, a local study conducted in Catalonia [22] reflected that the lack of knowledge and specific training focused on the identification of at-risk alcohol use, hinders the implementation of the screening and treatment of these patients.

On the other hand, our findings show heterogeneity in the selection of the questionnaires used to quantify alcohol consumption in PC. This issue reveals the importance of disseminating validated questionnaires on alcohol screening in the PC setting. Because of its validity and reliability, WHO and PAPPS recommend using the AUDIT questionnaire. Nationwide, a Spanish version of AUDIT has been validated in several studies [23] [24]. Rubio [23] published the validation of this questionnaire in a sample of 326 PC patients. Similarly, Pérula [24] validated the AUDIT questionnaire in a population of 614 Spanish adult patients, establishing a score of six or more points for diagnosing alcohol dependence, with a sensitivity and specificity of 88.3% and 83.1%, respectively.

As our results point out, the number of health professionals who claim to be aware of the PAPPS recommendations contrasts with those who provide these preventive measures in their clinical practice. Health advice for responsible alcohol use is the best-known recommendation by PC professionals, in contrast with the lesser-known guideline involving referral to a specialist or drug dependence center. In light of these results, it would be valuable to strengthen and disseminate the referral criteria for patients with at-risk alcohol use in the PC setting.

Regarding the accomplishment of the PAPPS recommendations by healthcare professionals, our study shows that relatively few PC professionals follow the PAPPS guidelines on this topic. In this setting, Pérula [25] indicated that PAPPS improves healthcare quality in PC centers and encourages the promotion of health and disease prevention; however, this author highlighted the lack of preventive strategies provided by PC professionals, probably due to the high-pressure working environment of healthcare providers.

In the overall results regarding clinical practice performed by healthcare professionals, the low level of systematic examination on alcohol consumption in PC is remarkable, even though this practice is one of the key preventive measures in alcohol use [26]. Babor [27] established this issue in the Brief Intervention guidelines published by WHO. This author indicates that PC professionals are in a privileged position to identify and intervene in those patients with at-risk alcohol use. Similarly, Fernández [28] highlighted the important role that PC professionals play in the follow-up of patients with alcohol use, mainly due to its accessibility and the leading position to promote alcohol prevention strategies.

Currently, several studies [29] [30] [31] [32] [33] demonstrate that healthcare advice can lead to a significant and lasting reduction in alcohol use. Furthermore, Ballesteros [29] showed in his meta-analysis of Spanish PC articles the efficacy of medical advice on reducing alcohol use. In this regard, the multi-center studies of Altisent [30] and Córdoba [31], conducted in 1997 and 1998, respectively, noted that alcohol prevention strategies are effective for reducing alcohol intake. Likewise, Bertholet [32] concluded that brief interventions are effective in reducing alcohol use at 6 and 12 months of follow-up, and even in longer periods.

In relation to the type of healthcare professionals who conduct their clinical practice in the PC setting, our study evidenced that nurses play an important role in the dissemination of preventive recommendations on alcohol use. There are previous studies focused on the effect produced by the nursing assessment on alcohol use; Mertens [33] described the short-term efficacy of screening and brief intervention implemented by nurses in a clinical trial conducted with Africans. Similarly, in a systematic review from 2014, Joseph [34] showed the effectiveness of nurse-conducted brief interventions on the reduction of alcohol use. Therefore, it is important that all PC professionals, including family physicians and nurses, become aware of this issue and participate in the screening and brief intervention involving the whole PC team [35].

Both level of training and attitude of PC professionals are essential in the approach to alcohol use. Anderson [36] indicated in his trial conducted in five European countries that training is one of the main strategies in the acquisition of a positive attitude towards alcohol management. In this line, Rosario [37] exposed that those professionals with a better attitude show a better approach to patients with at-risk alcohol use. Hence, the detection of how well PC professionals are trained in this issue, which has not been previously analyzed nationwide, is another example of the original contributions of this study. We have detected a high percentage of PC professionals who have no received specific training on alcohol management. Thus, it would be important to disseminate our results in order to promote specific training on alcohol use in the residency programs. Likewise, our outcomes should also be considered for Continuing Medical Education (CME) programs in the PC setting, since the level of specific training on this topic in older professionals and non-trainers is deficient.

One of the difficulties related to the assessment of knowledge, attitude, and preventive practices of professionals in this field lies in the absence of validated questionnaires. Consequently, surveys designed by expert professionals have been used to conduct our study. These questionnaires can serve as a basis for subsequent international research; thus, the current situation in other countries can be compared with the results obtained in this study. The mixed nature of the survey population, secondary to the application of different methods of recruitment, could be also considered a limitation of this study. However, the use of different methods of recruitment has allowed us to obtain a larger sample size.

In addition, another limitation presented in our study is the low response rate of healthcare professionals, as well as the possible screening bias, due to the fact that the questionnaire responses were voluntary; therefore, those PC professionals who were most motivated in this area were most likely to respond. This issue could modify the true prevalence of knowledge, attitude, and preventive practices of professionals in the approach to alcohol. In order to analyze the representativeness of the sample regarding the study population, we compared our data according to age and sex with those published by the Spanish Medical College Organization (MCO) [38] in 2015. According to the MCO, there were 54.2% of female family doctors in Spain, a lower percentage than in our study (62.9%); therefore, we can assume an overrepresentation of women. As we found that the prevalence of clinical practice addressing alcohol use was higher among women, we can conclude that the general level of practice was overestimated. With respect to age, a greater proportion of the participants in this study were family doctors. Considering a higher level of knowledge and practice observed among the older groups of professionals, an overestimation of the global prevalence of knowledge and practices of alcohol management should be suspected. Nonetheless, the sample size might be representative of PC health care professionals, since over 95% of PC professionals have their clinical practice in the NHS.

## Conclusions

In conclusion, this nationwide study reveals the current situation about the knowledge, attitude, and preventive practices of the Spanish PC health care professionals in the approach to alcohol use, based on the recommendations from PAPPS, a reference program in Spain and other Spanish-speaking countries. Our findings reveal a lack of knowledge and specific training on alcohol management, as well as a low level of preventive practice on alcohol use in PC. Due to the importance and magnitude of this healthcare issue in Spain, our results should be considered by scientific societies and policy makers in order to promote the implementation of alcohol prevention strategies in the PC setting.

## Supporting information

S1 Table Sociodemographic and occupational characteristics of professionals surveyed. (DOCX)

S2 Table Knowledge of PC providers on the approach to alcohol use according to sociodemographic and occupational characteristics. (DOCX)
