# Supplementary material for: Knowledge, attitudes and preventive practices of primary health care professionals towards alcohol use: A national, cross-sectional study
Source: PLoS One. 2019 May 13;14(5):e0216199. doi: 10.1371/journal.pone.0216199 (PMC6513087; doi:10.1371/journal.pone.0216199)
Supplement: S1 Table — (DOCX) [file pone.0216199.s001.docx]

S1 Table. Sociodemographic and occupational characteristics of professionals surveyed

| Characteristics of professionals | n (%) | | CI 95% |
| --- | --- | --- | --- |
| Sex  Male  Female  Age  Less than 35  36-45  46-55  56 or more  Type of professional  Family Physician  Nurse  Medical Resident  Resident Trainer  Yes  No  Membership to Scientific Societies  semFYC  SEMERGEN  SEMG  ASANEC  Other  Membership to specific programs: PAPPS * | | 653 (37.1)  1107 (62.9)  475 (27.2)  432 (24.7)  426 (24.4)  415 (23.7)  1.330 (75.6)  220 (12.5)  201 (11.4)  588 (33.4)  1172 (66.6)  1117 (63.5)  472 (26.8)  79 (4,5)  21 (1.2)  71 (4.0)  456 (25.9) | 34.8-39.4  60.6-65.2  24.9-29.1  22.5-26.6  22.2-26.2  21.6-25.6  73.5-77.6  10.9-14.1  9.9-12.9  31.2-35.6  64.4-68.8  61.2-65.7  24.7-28.9  3.5-5.5  0.7-1.7  3.1-5.0  23.8-28.0 |

* Program of Preventive Activities and Health Promotion (semFYC); 95% CI: 95% confidence interval
