# Supplementary material for: Knowledge, attitudes and preventive practices of primary health care professionals towards alcohol use: A national, cross-sectional study
Source: PLoS One. 2019 May 13;14(5):e0216199. doi: 10.1371/journal.pone.0216199 (PMC6513087; doi:10.1371/journal.pone.0216199)
Supplement: S2 Table — (DOCX) [file pone.0216199.s002.docx]

S2 Table. Knowledge of PC providers on the approach to alcohol use according to sociodemographic and occupational characteristics

|  | |  | |  | | | | Sociodemographic and occupational variables | | | | | | | | | | | | | | | | | | | | | | |  | |
| --- | --- | --- | --- | --- | --- | --- | --- | --- | --- | --- | --- | --- | --- | --- | --- | --- | --- | --- | --- | --- | --- | --- | --- | --- | --- | --- | --- | --- | --- | --- | --- | --- |
| Knowledge of PC professionals | | **Age** | | | | | | | | |  | | **Sex** | | | |  | | **Profession** | | | | | |  | | **Resident trainer** | | | |  | |
|  |  | **Less than 35**  **n (%)** | | **36-45**  **n (%)** | | **46-55**  **n (%)** | | | **56 or more**  **n (%)** | | **P value** ^a^ | | **Men**  **n (%)** | | **Women**  **n (%)** | | **p value** ^b^ | | **Physicians**  **n (%)** | | **Residents**  **n (%)** | | **Nurses**  **n (%)** | | **p value** ^b^ | | **Resident trainer**  **n (%)** | | **Not resident trainer**  **n (%)** | | **p value** ^b^ | |
| Alcohol as a risk factor | 312 (65.7) | | 261 (60.4) | | 248 (58.2) | | 212 (51.1) | | | <0.001 | | 391 (60.2) | | 642 (58.4) | | 0.242 | | 118 (54.1) | | 138 (66.3) | | 777 (58.8) | | 0.033 | | 351 (60.1) | | 682 (58.6) | | 0.290 | |  |
| Standard Unit Concept | 224 (47.2) | | 221 (51.2) | | 221 (51.9) | | 249 (60) | | | 0.001 | | 351 (54.1) | | 564 (51.3) | | 0.143 | | 107 (49.1) | | 88 (42.3) | | 720 (54.5) | | 0.003 | | 343 (58.7) | | 572 (49.1) | | <0.001 | |  |
| Concept of alcohol risk consumption applied to men | 187 (39.4) | | 215 (49.8) | | 227 (55.3) | | 244 (58.8) | | | <0.001 | | 328 (50.5) | | 545 (49.6) | | 0.369 | | 125 (57.3) | | 69 (42.3) | | 720 (54.3) | | <0.001 | | 333 (57) | | 540 (46.4) | | <0.001 | |  |
| Concept of alcohol risk consumption applied to women | 123 (25.9) | | 143 (33.1) | | 163 (38.3) | | 183 (44.1) | | | <0.001 | | 217 (33.4) | | 395 (35.9) | | 0.156 | | 77 (35.3) | | 54 (26) | | 481 (36.4) | | 0.014 | | 236 (40.4) | | 376 (32.3) | | 0.001 | |  |
| Concept of binge drinking applied to men | 148 (31.2) | | 141 (32.6) | | 176 (41.3) | | 165 (39.8) | | | <0.001 | | 240 (37) | | 390 (35.5) | | 0.282 | | 97 (44.5) | | 61 (29.3) | | 472 (35.7) | | 0.004 | | 235 (40.2) | | 236 (40.4) | | 0.006 | |  |
| Concept of binge drinking applied to women | 148 (31.2) | | 135 (31.3) | | 169 (39.7) | | 172 (41.4) | | | <0.001 | | 240 (37) | | 384 (34.9) | | 0.209 | | 94 (43.1) | | 59 (28.4) | | 471 (35.6) | | 0.006 | | 395 (33.9) | | 388 (33.3) | | 0.002 | |  |
| P value of total knowledge | |  | | p= 0.001^a^ | |  | | |  | |  | | p= 0.437^a^ | | | |  | |  | | p= 0.001^c^ | |  | |  | | p< 0.001^a^ | | | |  | |

^a^ P values obtained using the Mann-Whitney U test.

^b^ P values obtained using the Chi-square test

^c^ P values obtained using the ANOVA test
